# The association between seasonal influenza-like illness cases and foetal death: a time series analysis

**DOI:** 10.1017/S0950268818003254

**Published:** 2018-12-03

**Authors:** I. S. Rasmussen, L. H. Mortensen, T. G. Krause, A-M. Nybo Andersen

**Affiliations:** 1 Section of Epidemiology, Department of Public Health, University of Copenhagen, Copenhagen, Denmark; 2 Department of Clinical Microbiology, Herlev and Gentofte Hospital, University of Copenhagen, Herlev, Denmark; 3 Methods and Analysis, Statistics Denmark, Copenhagen, Denmark; 4 Department of Infectious Disease Epidemiology and Prevention, Statens Serum Institut, Copenhagen, Denmark

**Keywords:** Foetal death, influenza (seasonal), maternal infection, seasonality

## Abstract

It has been reported that foetal death follows a seasonal pattern. Influenza virus infection has been postulated as one possible contributor to this seasonal variation. This ecological study explored the temporal association between the influenza activity and the frequency of foetal death. Time series analysis was conducted using weekly influenza-like illness consultation proportions from the Danish sentinel surveillance system and weekly proportions of spontaneous abortions and stillbirths from hospital registers from 1994 to 2009. The association was examined in an autoregressive (AR) integrated (I) moving average (MA) model and subsequently analysed with cross-correlation functions. Our findings confirmed the well-known seasonality in influenza, but also seasonality in spontaneous abortion. No clear pattern of seasonality was found for stillbirths, although the analysis exposed dependency between observations. One final AR integrated MA model was identified for the influenza-like illness (ILI) series. We found no statistically significant relationship between weekly influenza-like illness consultation proportions and weekly spontaneous abortion proportions (five lags: *P* = 0.52; 11 lags: *P* = 0.91) or weekly stillbirths (five lags: *P* = 0.93; 11 lags: *P* = 0.40). Exposure to circulating influenza during pregnancy was not associated with rates of spontaneous abortions or stillbirths. Seasonal variations in spontaneous abortion were confirmed and this phenomenon needs further investigation.

## Introduction

Foetal deaths have wide-reaching psychological and social consequences for the expecting parents. Despite substantial interest in identifying the causes of foetal deaths, most cases remain unexplained [1, 2]. Hoyert and Gregory estimated that 30% of foetal deaths occurring at 20 weeks of gestation or later were classified as ‘foetal death of unspecified cause’ [3] and for miscarriages, the unexplained proportion is even larger.

Several studies have indicated that seasonal variation in spontaneous abortions and stillbirths exists [4–6]. Seasonal variation in spontaneous abortions has recently been demonstrated by Bruckner *et al*. on Danish register data [7]. The existence of seasonal variation in adverse birth outcomes may expose aetiologic factor(s) behind these. Influenza is known to follow a seasonal pattern with peaks during winter time, and it has been suggested that maternal influenza virus infection plays an aetiological role in foetal death, but the findings of current studies are inconsistent [8–11]. Transplacental transmission of influenza virus from mother to the embryo or foetus has been shown to occur, but is expected to be rare, so if a causal effect exists [12–14], it is most likely to work through a different mechanism, although we have shown that maternal fever was unrelated to foetal death in the same study population under evaluation here [15].

Few of the existing studies that examine the association between maternal influenza and foetal death consider the seasonal variation of both series in their choice of methodology. Zeger and colleagues have argued that methods that fail to account for autocorrelation in both series will fail to produce valid inference [16]. Since several studies and initiatives point towards an association between maternal influenza virus infection and foetal death [9, 17], and the fact that the suggested seasonal variation in foetal death should be taken into account in the statistical analysis in order to obtain valid inferences, we decided to apply an ecological time series method that accounts for dependency between observations and minimises confounding by examining the association between the influenza activity and two adverse birth outcomes: spontaneous abortion and stillbirth.

## Methods

### Data sources

Foetal death was defined by two outcomes: spontaneous abortions and stillbirths. Spontaneous abortion was defined as a non-deliberate foetal death of an intrauterine pregnancy before 22 completed gestational weeks whereas stillbirth was defined as any antepartum or intrapartum death of a foetus after the 22nd completed gestational week. All live births, spontaneous abortions and stillbirths to mothers with an estimated last menstrual period (LMP) in the period 14 February 1994 until 31 December 2009 in Denmark were included. Information was drawn from the National Patient Registry and the Medical Birth Registry using a unique personal identification number (CPR). The quality of the register has been described elsewhere [18, 19]. Both outcomes were coded according to ICD-10. Ectopic pregnancies, molar pregnancies and induced abortions were excluded from the dataset at the start. The time scale used in the study was calendar weeks of LMP. Weekly fetal death proportions were calculated by dividing the number of conceptions that ended in spontaneous abortions and stillbirths, respectively, by the total number of conceptions. Pregnancies were classified in conception-cohorts to account for varition in conceptions during the year. The influenza data were retrieved from the Danish sentinel surveillance system [20]. The surveillance system is based on weekly reports from on average 120 general practitioners all over Denmark and is an optional reporting system. The influenza-like illness case definition is sudden onset of fever, muscle ache and upper respiratory airway symptoms [21]. Weekly influenza-like illness proportions were calculated by dividing weekly number of patients with influenza-like illness by the total weekly number of consultations in general practice. These weekly influenza-like illness proportions were calculated for all weeks between week 7, 1994 through week 52, 2009. Influenza prevalence is not collected consistently for weeks outside the influenza period, which runs from the 40th calendar week of the year to the 20th calendar week of the subsequent year, and proportions were estimated by linear interpolation for most calendar weeks outside the influenza period (i.e. week 20–40 of a calendar year) and for calendar weeks with missing values.

### Ethics

According to Danish legislation, no ethical approval is needed for register studies based on completely anonymised data. The register linkage was approved by Statistics Denmark.

### Statistical analysis

Time series analysis can be applied when data are a sequence of observations arranged according to time and observations are expected to be dependent over time, i.e. autocorrelated. To be certain that the series were not white noise series, Ljung–Box statistics (*χ*^2^ tests) were performed [22]. In this study Box and Jenkins’ autoregressive (AR) integrated (I) moving average (MA) model (ARIMA) was used to pre-whiten the data [23]. The ARIMA model is able to handle the three components in time series: *p* is the order of the AR parameters, *d* is the difference order when the series is non-stationary and *q* is the order of the MA parameters. An ARIMA model was fitted to the dependent series, in this case, weekly proportions of influenza-like illness patients.

The first step in the Box–Jenkins approach was to determine whether the influenza-like illness series (hereafter influenza) was stationary. The order of differencing was determined by plotting the raw data in a time series plot and considering the graphical display of the autocorrelation function (ACF). A stationary series will fluctuate around a well-defined mean value and the ACF will rapidly converge to 0. If the data were determined to be non-stationary the trend was removed by differencing the series.

By examining the ACF, partial ACF (PACF) and inverse ACF (IACF) the preferred ARIMA models for the series were identified. The PACF indicates the preferred order of the AR part whereas the ACF indicates the preferred order of MA part. The fit of the preferred models was checked separately by the *t* tests of white noise and Akaike information criterion (AIC) was used to determine which model best described the observed influenza series [24]. When the final model is fitted to the series the residuals should not exhibit autocorrelation.

A pre-whitened cross-correlation function (CCF) was employed to identify the association between the influenza series and each of the two foetal death series. The CCF determined the correlation between the two series at both lags and leads [22, 25]. The applied regression model was in addition regressed on the absolute number of patients with influenza-like illness to ensure that the denominator size did not drive the variation.

## Results

### Descriptive statistics

Table 1 provides descriptive statistics of the time series, which includes observations from 1994 to 2009. The values in the table include the interpolated values of the influenza information. Information on influenza-like illness patients and number of consultations was unavailable in 291 weeks of the included 828 weeks, hence these weeks’ proportions were estimated by interpolation. In total, 13.3% of all conceptions ended in spontaneous abortions and 0.48% of all conceptions ended in stillbirths in the studied period.

**Table 1.** Descriptive statistics of the included time series

Time-series	*N* weeks^a^	Mean (s.d.)	Min.	Max.
Influenza-like illness patients per 100 consultations	828	1.29 (1.25)	0.06	10.05
Spontaneous abortions per 100 conceptions	828	13.33 (1.02)	10.62	16.58
Stillbirths per 100 conceptions	828	0.48 (0.20)	0.07	1.36

All registered pregnancies in Denmark, 1994–2009, and sentinel surveillance of influenza from Statens Serum Institut, Denmark, 1994–2009.

291 of the 828 weeks were estimated by linear interpolation.

a Includes calendar week 7, 1994–calendar week 52, 2009.

The Ljung–Box test confirmed that the influenza series, spontaneous abortion series and the stillbirth series contained some predictable patterns (1–24 lags: *P*-values <0.01). This means, for the foetal death series, that dependent of the week of the mother's LMP the foetuses have different risks of ending as foetal death. This was also the case when the Ljung–Box test was performed based on the absolute number of the patients with influenza.

**Fig. 1.**
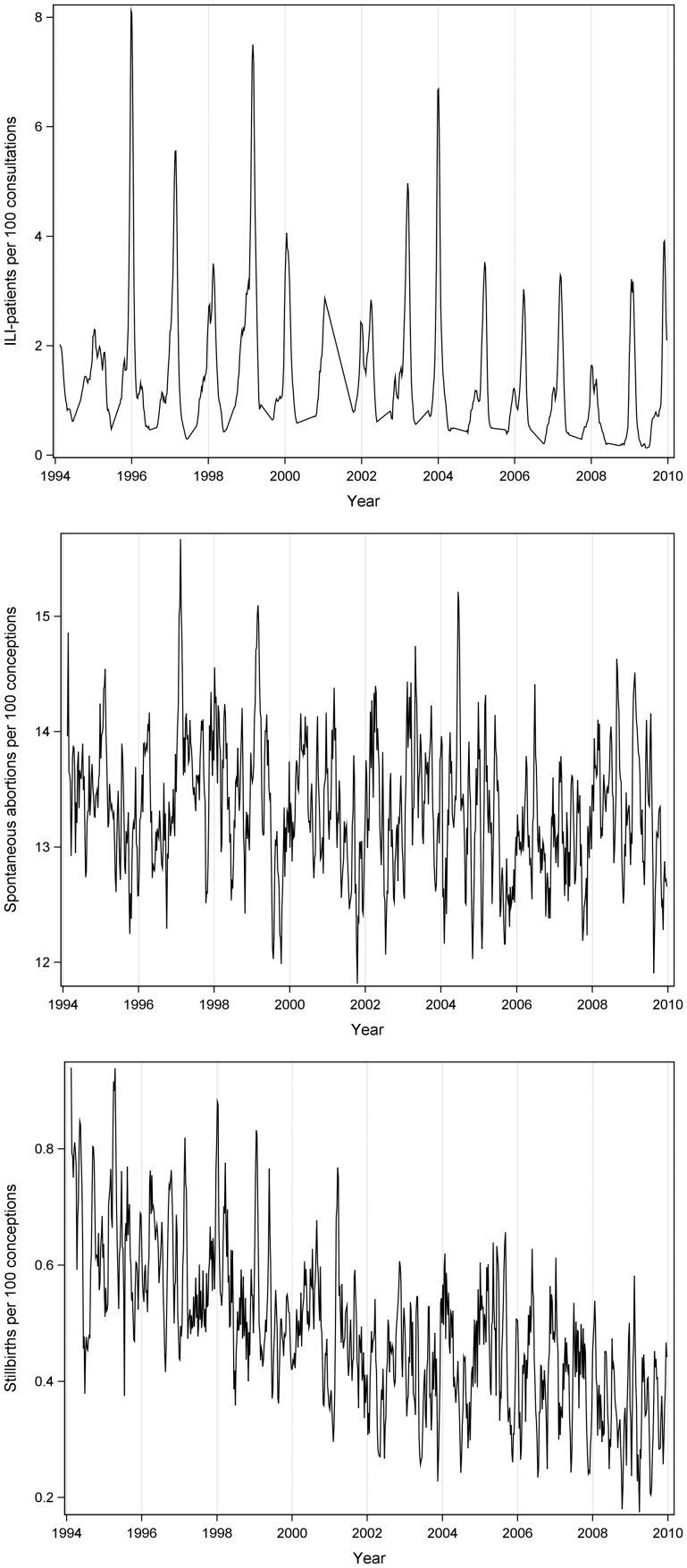
Four-week MA of weekly proportions of: influenza-like illness patients reported by general practitioners, spontaneous abortions and stillbirths occurring over the period of 1994–2009.

Figure 1 displays smoothed plots of the behaviour of spontaneous abortions and stillbirths per 100 conceptions together with a smoothed influenza series for the period of 1994–2009. The smoothed data, i.e. 4-week averages, are merely used for visual presentation. The plots showed the expected clear seasonal variation in the influenza series with peaks around the winter months. We also found a seasonal pattern for the spontaneous abortion with drops in calendar weeks 40–45, indicating that pregnancies of the mother with LMP period in October and the beginning of November are less likely to end in spontaneous abortion. The mean monthly number of spontaneous abortions has not changed across the studied period, which means that the series is fairly stationary. No visible seasonal pattern was seen for the number of stillbirths, but a notable decline in number of stillbirths was observed from 1994 to 2009.

### Pre-whitening/ARIMA-fitting

**Fig. 2.**
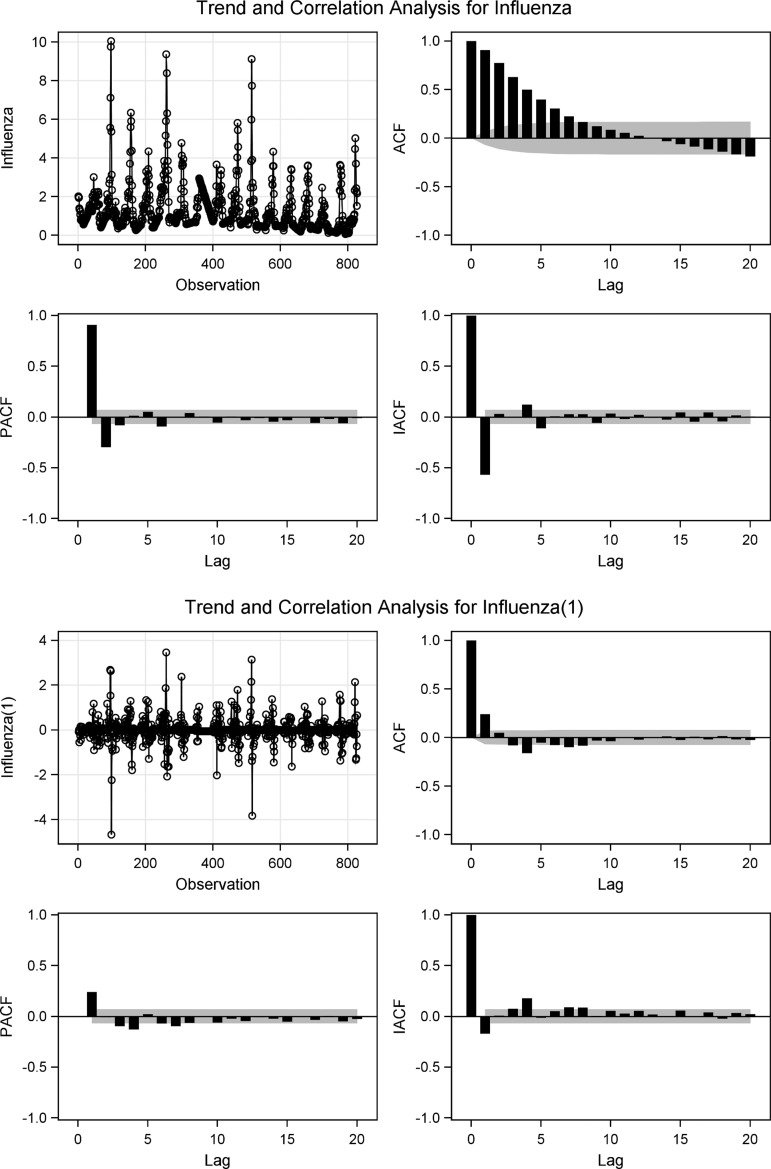
Behaviour, ACF, PACF and IACF of the non-stationary and stationary influenza series. The four lower plots represent the stationarised influenza series’ (influenza(1)) behaviour, ACF, PACF and IACF.

The temporal dependence structure in the influenza series is described in ACF and PACF plots (Fig. 2). According to the ACF-plot presented in Figure 2, the influenza series showed a non-stationary mean, thus we found it necessary to stabilise the series by differentiating the series once. The influenza series was therefore differenced once in order to estimate the influenza series’ mean, variance and covariance over time (i.e. the assumption of stationarity), *I*(*d*) = 1. All further statistical procedures were performed on the transformed influenza series. The transformed influenza series is in Figure 2 indicated ‘Influenza(1)’.

A total of four different ARIMA models were suggested by the distribution characteristics to fit the influenza series: ARIMA(3, 1, 3), ARIMA(3, 1, 4), ARIMA(4, 1, 3) and ARIMA(1, 1, 4). When the models were compared by AIC score, ARIMA(1, 1, 4) had the best fit verified by the smallest AIC score (AIC = 1204.8) compared with the other ARIMA models with AIC between 1215.24 and 1219.48. The ACF and PACF of the suggested models were also compared. The residuals of the preferred model were uncorrelated for all lags based on the ACF plot, the partial correlation function plot and autocorrelation check for residuals (lag 12: *χ*^2^ = 9.83, *P* = 0.20; lag 18: 12.80, *P* = 0.46). The parameter estimates of ARIMA(1, 1, 4) were significant (*P* < 0.01). The ARIMA(1, 1, 4) revealed the best fit for the influenza series.

### Cross-correlation analysis

We filtered the ARIMA(1, 1, 4) with the dependent influenza series, to reduce the residuals to white noise. The two outcome series: spontaneous abortion and stillbirth were similarly filtered with the ARIMA(1, 1, 4). The filtered influenza series was cross-correlated with the two filtered response series: spontaneous abortion and stillbirth.

**Fig. 3.**
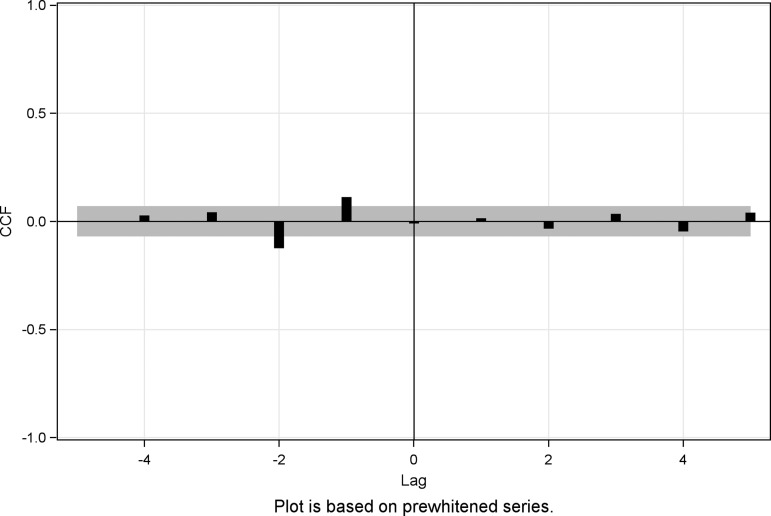
CCF plot of the pre-whitened influenza series and the spontaneous abortion series, Denmark, 1994–2009.

**Fig. 4.**
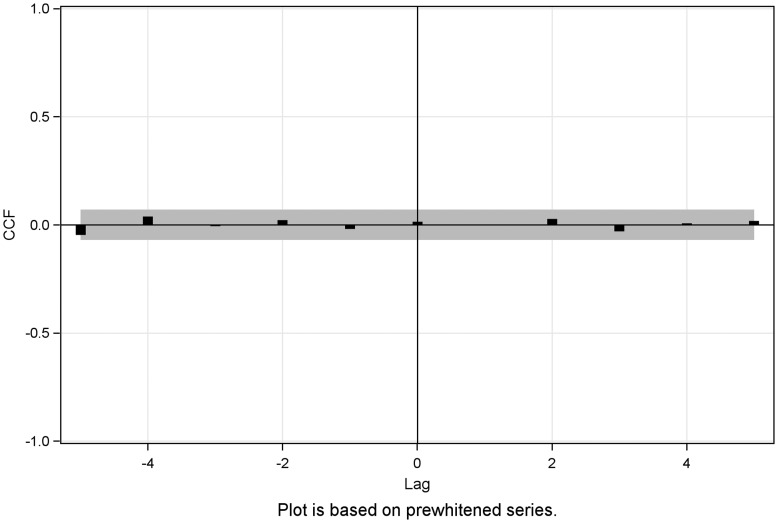
CCF plot of the pre-whitened influenza series and the stillbirth series, Denmark, 1994–2009.

The CCF between the influenza series and the spontaneous abortion was estimated and is illustrated in Figure 3. We found no significant correlation between the influenza series and the spontaneous abortion series (five lags: *P* = 0.52; 11 lags: *P* = 0.91). We did not find any significant correlation between either the influenza series or the stillbirth series, estimates were insignificant at all lags (five lags: *P* = 0.93; 11 lags: *P* = 0.40). The CCF between the influenza series and stillbirth series is illustrated in Figure 4.

## Discussion

Our findings suggest that spikes in influenza are uncorrelated with spikes in both spontaneous abortions and spikes in stillbirths. However, the results of this study confirm the existence of autocorrelation, including seasonal variation, for both spontaneous abortions and stillbirths in 1994–2009 in Denmark. Our results are in line with a related study by Fell *et al*. who concluded that stillbirth rates and perinatal death rates were unassociated with gestational exposure to influenza circulation during late pregnancy [26]. The current study is the only study besides Fell *et al*.’s study that has applied time series analysis to examine the association between exposure to influenza circulation during pregnancy and stillbirths. Fell *et al*. did, however, not include spontaneous abortions as an outcome in their study.

This study uses an ecological time series design, which incurs at least two problems [27]. The first problem is the potential risk of change of disease classification [27]. This was not a concern in this study, as neither the disease classification of influenza nor foetal death was changed through the 15 years studied period. Individual-level confounders are often not a problem in time series studies, thus controlling for individual socio-demographic covariates do not make sense unless they co-vary with exposure and outcome [27], which is unlikely in our view since recognised individual-level risk factors of foetal death are unlikely to exhibit seasonality, despite a change in maternal socio-demographic characteristics across the observations and time. Buckles and Hungerman have suggested that seasonality in foetal death reflects inherent differences in personal attributes or family background [28]. We were not able to reject this hypothesis, which must be considered a limitation of the study. Confounding can moreover occur as a result of time correlated exposures, like pollutants or other infectious agents. We cannot rule out that other environmental exposures could be at play.

The choice of the preferred ARIMA model was based on the AIC-test together with a check of the residuals. The AIC scores mean nothing on their own. The AIC-scorers of the four models were comparable, although they pointed towards a preference for the preferred ARIMA. Comparing the AIC-scores suggested that increasing the number of AR parameters estimated worsened the fit of the model. The residuals of the final model indicated a well-fitted model as suggested by the AIC-scorers.

Information on foetal deaths and live births was obtained from the national registers, which provide longitudinal registration of conceptions for the whole population [19, 29]. A limitation of data is that we have only included clinically recognised pregnancies. Bloom-Feshbach *et al*. found birth rates declined during the 1918 pandemic in the United States, Denmark, Sweden and Norway suggesting that missing births were attributable to excess first trimester miscarriages [30]. Our results do not support this result. If maternal influenza virus infection had a negative effect on the foetus in a very early stage, the registered pregnancies would result in a delayed seasonal pattern. The findings were based on proportions of foetal death (e.g. spontaneous abortions or stillbirths) per 100 conceptions *without* induced abortions, molar pregnancies and ectopic pregnancies. The proportions did consequently not include the events that compete with the outcome of interest to remove foetuses from all of the conceptions. The calculation of the proportions entails a problem since a portion of conception that potentially could have ended in spontaneous abortion or stillbirth was excluded, the total number of conceptions is likely underestimated. This would not be a problem if the number of conceptions conceived and the number of induced abortion, molar pregnancies and ectopic pregnancies were constant. Bruckner *et al*. recently showed that there exists a seasonal pattern in induced abortions, which contradicts the perception of a constant number of induced abortions within a calendar year [31]. The exclusion of the induced abortions from the dataset could lead to deceptive seasonality in foetal death due to selection bias. The seasonality in the induced abortions could also constitute a problem if the proportions were calculated based on all conceptions due to the fact that induced abortion is a competing risk for foetal death. Bias as a result of this seasonality in induced abortion is not possible to rule out in ecological studies, regardless of the construction of the dataset. We were not able to separate *ante*-partum stillbirths from *intra*-partum stillbirths in the analyses despite potential differences in risk factors and aetiology [32], we ought to consider if this could be a problem. Antepartum stillbirths and spontaneous abortions both refer to the death of foetus occurring before the initiation of labour but at different times during pregnancy, and we expect that antepartum stillbirths are more likely to behave like spontaneous abortions than intrapartum stillbirths are. Intrapartum stillbirth defines late foetal death *during* labour. Rates of intrapartum stillbirths are assumed to reflect the quality of care in labour [32] and will be randomly distributed at best in developed countries like Denmark, thus we do not believe the inclusion of both antepartum stillbirths and intrapartum stillbirths bias our results. The study population covered all clinically registered pregnant women in Denmark over a period of 15 years, thus minimising the selection bias.

The exposure variable, proportions of patients with influenza-like illness, was a proxy variable for the influenza activity across the years in Denmark. The proxy measure was meant to describe how the distribution of maternal influenza virus was across the year and across the time period of interest. The group of general practitioners who reports to the sentinel surveillance system is volunteers, and although Statens Serum Institut reports that the reporting system is random, there is no assessment of the general practitioners’ geographical placement. A limitation of this system is that there is a possibility that the majority of the volunteering practitioners could have been placed in either rural or urban places, even though their reporting is meant to reflect the influenza virus distribution across both types of geographic areas. Another limitation of this system is the way the diagnosis is suggested. The general practitioners base the diagnoses of influenza virus solely on symptoms. No specimen tests are required, although this approach is the only one which gives guarantee for correct diagnosis. Most upper respiratory viruses, e.g. respiratory syncytial virus and rhinoviruses, can cause cold-like symptoms or even influenza-like symptoms. Seasonality is also a common phenomenon among most upper respiratory viruses [33]. Our findings indicate no association between influenza virus and foetal deaths, and we cannot reject that this non-association accounts for other upper respiratory diseases with influenza-like symptoms and behaviour. Furthermore, we did not include information on which influenza types or subtypes that were circulating in the time period. Therefore, we cannot exclude that there could be an association with circulation of certain types and subtypes and foetal deaths. Another inaccuracy is that the influenza-like illness case proportions calculated were based on consultations in the whole population and not only among pregnant women, thereby representing a proxy measure of circulation of influenza within the whole population. Thus, the proportions included infected children, which represent a group that is more often affected by influenza viruses than adults in particular with type B. The proportions also included elderly people infected with influenza virus. It is known that elderly individuals are more likely to get medical complications in connection with influenza infection compared with other people who get infected with influenza virus. The hypothesis and justification of using this proxy measure in this study are, however, that number of infected individuals in the population will reflect the number of infected pregnant women. There are no clear indications that the proportions of influenza-infected pregnant women differ from the rest of the population. We have no reason to believe that pregnant women's influenza proportions will not follow the influenza proportions of the general population. We therefore expect that the proportion of influenza-infected pregnant women will follow the influenza burden in society. However, previous studies suggest that influenza is more likely to cause severe illness in pregnant women than in women of reproductive age who are not pregnant [34]. Authors who have examined the quality of the sentinel surveillance system in Europe have concluded that the clinical data reported by sentinel physicians are a valid indicator of influenza virus activity [35]. The findings by Paget *et al*. showed a good match between the clinical sentinel data and laboratory reports of influenza virus collected by sentinel general practitioners [35]. We are therefore confident that our clinical sentinel data are valid for assessing influenza activity, which is why we believe that adding virological laboratory information would not change our results.

The proportions between numbers of consultations and numbers of patients with influenza-like illness can lead to artificial high proportions in holidays, since the number of planned consultations is lower, and the number of acute patients (e.g. with influenza virus) is higher. The influenza variable was also arranged and modelled as the absolute number of patients with influenza-like illness to account for this type of bias. This did not change the results markedly. The interpolation of the missing data, especially outside the influenza season could constitute a problem. The actual influenza virus activity was not known in these weeks and the calculated influenza proportions in these missing weeks do not necessarily reflect the true influenza-like illness activity in Denmark. The influenza A/H1N1 pandemic in 2009 began earlier than the normal seasonal cycle. Peaks in the time series caused by the H1N1-pandemic were, possibly and unintentionally, not a part of the dataset. Bias related to this issue was difficult to handle since influenza virus information was not available.

Until 2009/2010 free influenza virus vaccination has only been offered to pregnant women with certain chronic diseases. The vast majority of the cohort included in this study would therefore not have been offered or recommended the influenza vaccination by their general practitioner. Though, women, who were pregnant in the influenza season 2009/2010 might have been offered- and accepted the pandemic influenza vaccination. Influenza vaccination during pregnancy would tend to underestimate any association between influenza activity and the studied outcomes. As only a very small proportion of pregnant women would have been vaccinated in the study period this is not thought to constitute a major problem in the study.

In conclusion, our findings did not support the hypothesis that maternal influenza increases the risk of foetal death, even when the limitations of ecologic data are considered. The seasonal variation in foetal deaths remains thus to be explained.
